# Feasibility and acceptability of an mHealth, home-based exercise intervention in colorectal cancer survivors: A pilot randomized controlled trial

**DOI:** 10.1371/journal.pone.0287152

**Published:** 2023-06-22

**Authors:** Ann Marie Moraitis, Nathan B. Rose, Austin F. Johnson, Emily R. Dunston, Ignacio Garrido-Laguna, Paula Hobson, Kristin Barber, Karen Basen-Engquist, Adriana M. Coletta

**Affiliations:** 1 College of Nursing, University of Utah, Salt Lake City, Utah, United States of America; 2 Department of Health and Kinesiology, University of Utah, Salt Lake City, Utah, United States of America; 3 Department of Internal Medicine, Division of Oncology, University of Utah, Salt Lake City, Utah, United States of America; 4 Huntsman Cancer Institute at the University of Utah, Salt Lake City, Utah, United States of America; 5 Department of Behavioral Science, The University of Texas MD Anderson Cancer Center, Houston, Texas, United States of America; PhD, PLOS, UNITED KINGDOM

## Abstract

**Objective:**

To determine the feasibility and acceptability of an mHealth, home-based exercise intervention among stage II-III colorectal cancer (CRC) survivors within 5-years post-resection and adjuvant therapy.

**Methods:**

This pilot randomized controlled trial of a 12-week mHealth, home-based exercise intervention, randomly assigned CRC survivors to a high-intensity interval training (HIIT) or moderate-intensity continuous exercise (MICE) prescription. The following assessments were carried out at baseline and end-of-study (EOS): handgrip strength, short physical performance battery (SPPB), PROMIS physical function, neuropathy total symptom score-6 (NTSS-6), Utah early neuropathy scale (UENS), cardiopulmonary exercise testing, anthropometrics, and body composition via BOD POD, modified Godin leisure-time activity questionnaire. Feasibility, as defined by number of completed prescribed workouts and rate of adherence to individualized heart rate (HR) training zones, was evaluated at EOS. Acceptability was assessed by open-ended surveys at EOS. Descriptive statistics were generated for participant characteristics and assessment data.

**Results:**

Seven participants were included in this pilot study (MICE: n = 5, HIIT: n = 2). Median age was 39 years (1^st^ quartile: 36, 3^rd^ quartile: 50). BMI was 27.4 kg/m^2^ (1^st^ quartile: 24.5, 3^rd^ quartile: 29.7). Most participants had stage III CRC (71%, n = 5). We observed an 88.6% workout completion rate, 100% retention rate, no adverse events, and qualitative data indicating improved quality of life and positive feedback related to ease of use, accountability, motivation, and autonomy. Mean adherence to HR training zones was 95.7% in MICE, and 28.9% for the high-intensity intervals and 51.0% for the active recovery intervals in HIIT; qualitative results revealed that participants wanted to do more/work-out harder.

**Conclusion:**

An mHealth, home-based delivered exercise intervention, including a HIIT prescription, among stage II-III CRC survivors’ post-resection and adjuvant therapy was tolerable and showed trends towards acceptability.

## Introduction

It is estimated over 151, 000 individuals will be diagnosed with colorectal cancer (CRC) in 2022, resulting in nearly 8% of all new cancer cases [1]. Surgical excision is the only curative treatment for localized disease [2]. However, there are high recurrence rates (30% for stage II, 50–60% for stage III tumors) following surgery [3], with most occurring in the first five years [4]. Engagement in regular exercise among CRC survivors is safe and associated with improvements in morbidity and significant improvements in mortality [5–11]. According to the World Health Organization, exercise recommendations for adults is 150–300 minutes/week, or vigorous aerobic physical activity for 75–150 minutes/week [12]. Additionally, there is evidence to suggest higher levels of weekly exercise (e.g. ≥ 18 metabolic equivalent hours [MET-hrs.], a metabolic equivalent of 1 is equal to resting metabolic rate) may be linked with a 50% reduction in CRC recurrence [13]. Despite this, research indicates that the majority of CRC survivors do not engage in recommended levels of physical activity [14]. To date, the optimal PA delivery mode for CRC survivors is not clear [15]. Qualitative research in cancer survivors indicates a preference for integration of physical activity (PA) mHealth applications into survivorship care noting the value of guidance and recommendations from the health care team [16]. Additionally, mHealth delivery promotes self-engagement and the survivors ease of access, regardless of geographical location, to supportive care [17].

Exercise oncology was identified as one of three focus areas for the expansion of mHealth, along with initiation and self-management support and survivorship care delivery [13]. Theory driven mobile health technologies (mHealth) have been used in cancer survivorship to improve provider-patient communication and patient-driven engagement in their own cancer care [18]. Recently, mHealth platforms have emerged as a transformative mode of cancer care delivery that improves patient engagement, independence, and convenience/access [19]. Research indicates lack of time is the biggest barrier to exercise adoption [20,21]. A salient theme for exercise preferences among cancer survivors is home-based exercise [22]. Among CRC survivors, home-based methods have been shown to increase regular engagement in exercise acutely and long-term [23,24]. However, there are limited mHealth, home-based exercise interventions [25]. Research indicates mHealth home-based delivered PA interventions show statistically significant improvements in the level of PA improvements, including increases in moderate-vigorous PA in cancer survivors [26]. and in health-related quality of life [27,28].

Moderate-intensity exercise is performed at 64–76% of age predicted maximal heart rate (220-age) [29]. An alternative time-efficient approach to moderate-intensity continuous exercise (MICE) is high-intensity interval training (HIIT). for example 4-minutes of exercise at 80–90% of age-predicted maximal heart rate, and low-intensity intervals or rest periods, which can provide the same dose of exercise (e.g. MET-hrs.) in less time due to the increase in intensity [30,31]. Previous work from cancer patients across all stages of the cancer care continuum indicates that health benefits of HIIT workouts are similar to MICE workouts [32]. Thus, a potential advantage of HIIT in CRC survivors is that it may facilitate increased initial participation in regular exercise by removing the barrier of total weekly time commitment. Among CRC survivors, only two studies, to our knowledge, have compared HIIT to MICE, and this was in a supervised, in-person setting [33,34]. Collectively results demonstrated feasibility and safety of supervised HIIT in stage I-IV CRC survivors at least one-month post-treatment [30]. Further, when compared to MICE, significant improvements in body composition and cardiorespiratory fitness were only observed with HIIT [33,34]. In summary, HIIT interventions remove the barrier of time and an mHealth, home-based delivery approach adopts two facilitators and preferences articulated by cancer survivors to foster sustained exercise engagement in survivorship. Thus, the purpose of this pilot randomized controlled trial was to determine the feasibility and acceptability of an mHealth, home-based delivered exercise intervention, including MICE and HIIT prescriptions, among stage II-III CRC survivors within five years post-resection and adjuvant chemotherapy. We hypothesized that the mHealth home-based delivered exercise prescriptions would be feasible and acceptable among CRC survivors.

## Methods

### Study design

**Fig 1:**
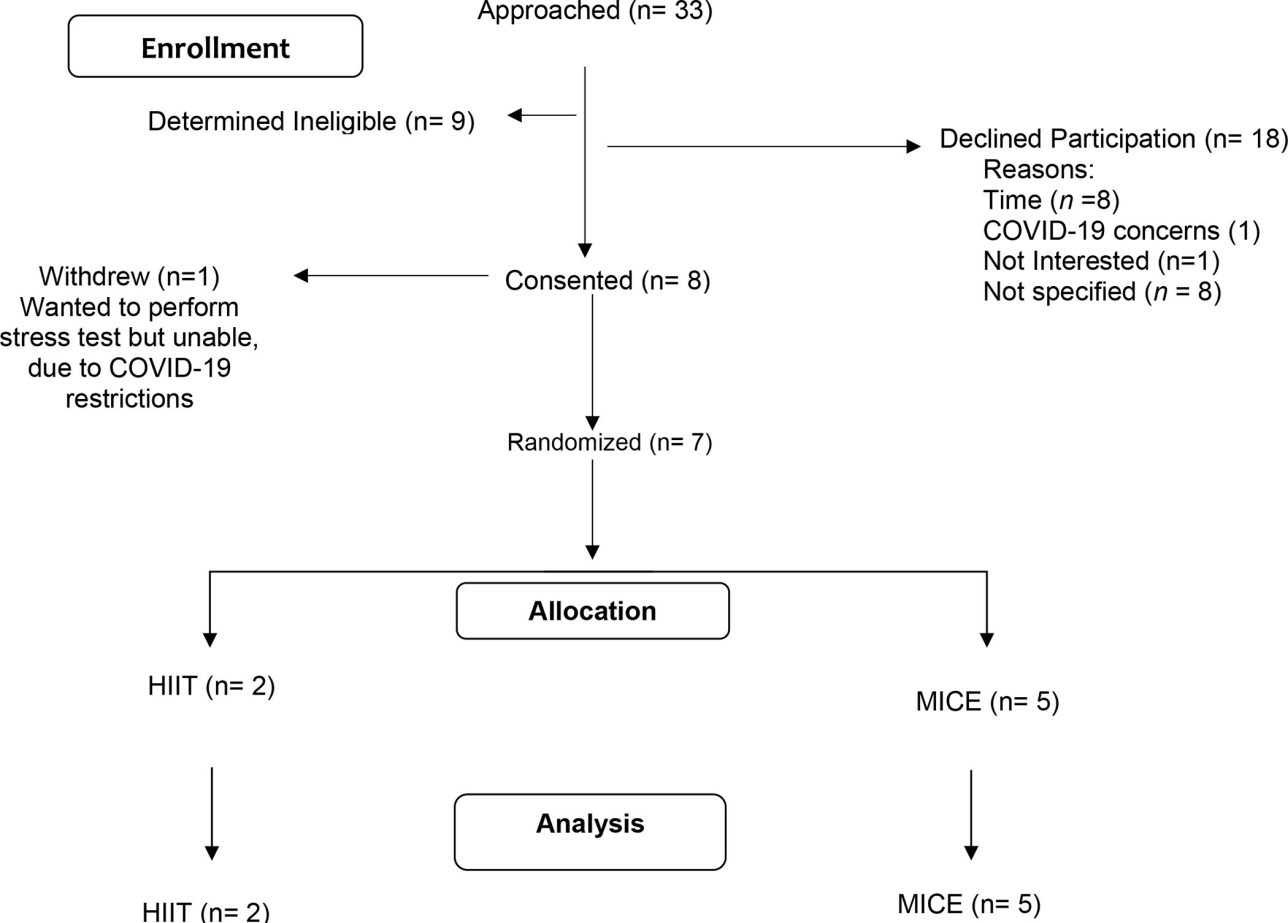
CONSORT diagram.

This parallel randomized controlled trial was a 12-week mHealth, home-based exercise intervention. Exercise was remotely monitored utilizing the Polar® A370 (Polar Electro: Kempele, Finland) fitness tracking device and Polar® H10 Heart Rate (HR) sensor, that is worn around the chest. Participants were randomized to either a high-intensity interval training (HIIT) or moderate-intensity continuous exercise (MICE) intervention that provided the same dose of exercise. REdCap was used to generate the random allocation sequence. Assessments occurred prior to the start of the exercise intervention (baseline) and upon completion of the intervention (end of study). Feasibility was assessed at end of study (EOS). All participants were instructed to continue their typical diet and medication regimens throughout the intervention. The study was approved by the University of Utah Institutional Review Board and registered in ClinicalTrials.gov (NCT04080414). Enrollment in the trial began in Fall 2019. Goal enrollment was 30 evaluable participants. Fig 1 provides the CONSORT diagram for the study.

### Participant recruitment and eligibility

Stage II-III CRC survivors less than five years post-resection and adjuvant chemotherapy were recruited from the gastrointestinal medical oncology clinics at the Huntsman Cancer Institute at the University of Utah. Other eligibility criteria included: age 19–75 years; currently engaging in less than 90-minutes of structured moderate- or vigorous-intensity aerobic exercise; no known cardiovascular, metabolic, or renal disease, and no signs/symptoms suggestive of cardiovascular, metabolic, or renal disease per the American College of Sports Medicine’s exercise pre-participation health screening questionnaire, and access to a computer or smart phone. Access to a computer or smart phone was necessary in order to download exercise training data from the Polar A370 device to the participant’s Polar Flow account, which was created by the study team. Patients were excluded if they had clinically evident recurrent disease, and/or a resting systolic blood pressure ≥ 140 mmHg and/or resting diastolic blood pressure ≥ 90 mmHg at the time of baseline testing, and/or functional limitations requiring a walker, scooter, or wheelchair.

### Assessment sessions

Baseline and EOS assessments were completed at a research center located on the University of Utah campus, which is adjacent to the health sciences/medical campus. The study team aimed to schedule assessments at times that aligned with standard of care visits with each participant’s gastroenterologist medical oncologist to minimize travel time and participant burden. A demographics questionnaire was administered at baseline. An exit survey with space for open-text response to expand on acceptability was included with the EOS assessment. All other procedures were followed for baseline and EOS assessments.

### Demographics questionnaire

This instrument inquired about age, race/ethnicity, marital status, education level, employment status, annual household income, and current smoking status.

### Exit survey

All participants completed a EOS assessment that contained space for open-text response to expand on acceptability of the exercise intervention and use of mHealth technology.

### Anthropometrics & body composition

Weight (kg) and height (cm) were measured using standard procedures [29]. Body composition was measured with air displacement plethysmography (BOD POD, COSMED USA, Concord CA) following standard procedures. Air displacement plethysmography was selected as opposed to other methods, such as dual-energy x-ray absorptiometry, due to availability of equipment in the research center.

### Cardiopulmonary exercise testing

The cardiopulmonary exercise test followed the modified Balke protocol [29]. The Balke protocol was used as opposed to the most widely used Bruce protocol because it is considered the more appropriate method for clinical populations and untrained individuals [29,35,36]. The ParvoMedics TrueMax 2400 Metabolic Measurement System (Parvomedics Inc, Sandy, UT) was used to assess peak aerobic capacity following standard procedures. A 12-lead electrocardiogram was included to measure heart rate (HR) throughout testing and monitor for safety purposes. Prior to initiation of the modified Balke protocol, a 2-minute warm-up was completed. Rating of perceived exertion, HR, and blood pressure were collected at the end of every stage. The participant continued the test until he/she felt unable to continue; the measurement at that point was considered the peak aerobic capacity and peak HR. All participants completed at least a 2-minute walking cool down.

### Peak heart rate estimation

Peak HR measured during each participant’s baseline cardiopulmonary exercise test dictated exercise intensity for that individual’s exercise prescription throughout the 12-week intervention. Data collection for this trial started in Fall 2019. After March 2020, due to COVID-19 research restrictions, we were unable to complete two baseline (and five EOS) cardiopulmonary exercise tests. For these cases, peak HR was estimated utilizing the age-predicted maximum HR equation developed by Fox and colleagues [37]. This equation was utilized, as opposed to other equations [38–40]. based on analysis of fit with measured peak HR data from this study. Briefly, Friedman’s ANOVA was used to determine if there were significant differences between measured and predicted Heart rates [38]. Statistically significant differences between predicted and measured values were not observed. Age-predicted maximum HR was decided because this prediction was closest to the measured values in this patient sample.

### Handgrip strength

Physical function assessment consisted of tests for muscular strength and physical performance. The handgrip strength test is a widely used marker of muscular strength, as such we included this procedure in our investigation. A portable, hydraulic dynamometer (Jamar 5030J1) was used following a static endurance handgrip strength protocol [41].

### Short physical performance battery (SPPB)

The short physical performance battery was used to assess physical function and performance. SPPB is widely used both in a research and clinical setting to evaluate physical function. This method consists of three components [42,43]: *1) Walking Speed-* Participants were instructed to walk four meters, at 0% incline, as quickly as possible [42]; *2) Standing Balance-* Participants were instructed to stand up with their feet in three different positions (side-by-side, tandem and semi-tandem) for 10 seconds each position [42]; *3) Sit-to-Stand Performance-* Participants were instructed to rise from a chair for five times consecutively [42].

### PROMIS physical function

The PROMIS Physical Function Short Form 6b complemented the objective measures of physical function. This version, 6b, was selected as opposed to other versions of the PROMIS Physical Function due to its strong reliability and minimal participant burden. The questionnaire consists of six items requiring a response on a scale from “without any difficulty” to “unable to do” for questions 1–4 and “not at all” to “cannot do” for questions 5 and 6. This questionnaire has an average reliability coefficient of about 0.95 [8].

### Neuropathy total symptom score-6 (NTSS-6)

Both subjective and objective measures were used to evaluate prevalence and change in CIPN signs and symptoms. The NTSS-6 is a widely used 15-item questionnaire inquiring about whether (yes or no) an individual is experiencing the specified feeling in his/her legs and feet. It has an internal consistency of α > 0.7 and an intra-class correlation coefficient of > 0.9 [8,44].

### Utah early neuropathy scale (UENS)

The UENS is a clinical examination focused on small fiber sensory function. The interrater reliability of UENS is 94% [45], and its diagnostic efficiency as measured by area under the curve is > 0.9.

### Modified Godin leisure time physical activity questionnaire (Godin)

The modified Godin questionnaire inquires weekly frequency (times per week and average number of minutes each time) of participation in strenuous, moderate, and mild aerobic exercise. This questionnaire has a reliability coefficient of 0.81, along with significant correlations with peak aerobic capacity [46].

### Intervention

#### Equipment and exercise protocol familiarization session

Following completion of baseline assessment procedures, both HIIT and MICE participants completed an Equipment and Exercise Protocol Familiarization Session pertaining to the activity tracking device and randomly assigned exercise intervention. Study personnel helped participants set up the Polar® A370 (Polar Electro Oy: Kempele, Finland) fitness tracking device and Polar® H10 HR sensor and demonstrated how to use the devices during exercise. The Polar®A370 is incorporated with a wrist-band heart rate monitor and compatible with the Polar H10 HR chest sensor. Participants were encouraged to wear the H10 chest sensor during each workout to obtain the most accurate measurement of HR; however, if a participant forgot to wear the chest sensor, HR was still recorded from the wrist sensor on the A370 device. Upon completion of the setup process, participants completed their first workout with the device, supervised by study personnel, on a treadmill. A study binder including written instructions of the information provided during the session was given to participants to take home and keep.

#### Mode of aerobic exercise for the intervention

Participants, in both groups, self-selected the mode of weight-bearing aerobic exercise (i.e., walking, jogging, elliptical, calisthenic exercises). Only weight-bearing activity was performed since the goal HR zones were based on peak HR measured during weight-bearing exercise (cardiopulmonary exercise testing on a treadmill).

### High-intensity interval training (HIIT)

The goal HIIT prescription consisted of four, 50-minute workouts per week. Each workout consisted of a 10-minute warm-up at 50–75% peak HR followed by five, 4-minute intervals at 85–90% peak HR with four minutes of active recovery at 50–75% peak HR following each 4-minute interval. This prescription was selected to facilitate an exercise dose of ≥18 MET-hrs./wk, based on previously literature supporting this exercise dose to reduce the risk of CRC recurrence [13].

### Moderate-intensity continuous aerobic exercise (MICE)

The goal MICE prescription was matched with HIIT in dose, and consisted of five, 60-minute workouts per week, at 50–70% peak HR. Both exercise prescriptions included a 4-week progression in time and intensity to goal exercise prescription.

### Monitoring exercise adherence

The Polar A370 device and Polar H10 HR chest sensor enables participants to monitor HR during each workout. The Polar®A370 device synchronized with the Polar Flow application on any smartphone device or computer. Participants were able to monitor their progress and view all exercise information with the Polar Flow application. The Polar Flow application synchronized with the Polar Coach application which allowed the study team to remotely monitor participants during the intervention. A member of the study team monitored intervention adherence weekly. If a participant was not adherent to the protocol for the week, a member of the study team called the participant and applied motivational surveying and problem-solving techniques to identify any challenges participants may be facing in adhering to the exercise prescription.

### Statistical analysis

Participant characteristics were evaluated with descriptive statistics and presented as either frequency and percentage or median and first and third quartiles. Feasibility of HIIT was assessed at EOS. We considered our intervention feasible based on both the number of prescribed workouts completed throughout the intervention, and the rate of adherence to the individualized exercise prescription throughout the intervention. Our goal was ≥75% of prescribed workouts completed among participants, and ≥75% of participants adhering to their individualized exercise prescription. Adherence was defined as completing or exceeding ≥70% of the workouts in a manner that is consistent with the exercise prescription. We defined being consistent with the exercise prescription as maintaining HR within all prescribed zones or ±5 beats per minute outside of each zone throughout the workout. Change from baseline to EOS was calculated for the following variables collected during assessment sessions by the total sample and by group: body weight, fat mass, fat-free mass, body fat percentage, handgrip strength, SPPB, PROMIS physical function score, NTSS-6 score, and UENS score. Peak aerobic capacity data was not analyzed due to the limited number of participants able to complete both a baseline and EOS test due to research restrictions from the COVID-19 pandemic. Assessment session data is presented as mean and 95% confidence intervals (CI). Assessment session data was compared to established cut-points for minimal clinically important differences for the following: hand grip strength (+5–6.5kg) [47,48], SPPB (> 1-point) [49]. All data were analyzed with SPSS, version 26 (Chicago, IL). EOS open-text responses were transcribed and analyzed using conventional qualitative content analysis [50], and MAXQDA to organize results (VERBI Software, 2019). Results were reviewed and discussed until consensus was reached among members of the research team. Main themes related to the acceptability of the mhealth, home-based intervention were extracted from open-text responses.

## Results

### Participant characteristics

Data for this trial was collected between November 2019 and February 2021. The target sample size was 30 evaluable participants; however, the trial was ended prematurely due to the impact of the COVID-19 pandemic on recruitment. A total of seven evaluable participants completed the trial (n = 2 in HIIT, n = 5 in MICE). A participant was considered evaluable if he/she completed baseline and EOS assessments. Median age of participants was 39 years (1st quartile: 36, 3rd quartile: 50), and median BMI was 27.4 kg/m2 (1st quartile: 24.5, 3rd quartile: 29.7). Most participants were diagnosed with stage III colorectal cancer (71%, *n* = 5). All participants were non-Hispanic, White, married, and the majority were employed while participating in the intervention (86%, *n* = 6). Table 1 provides more details related to participant characteristics.

**Table 1:** Participant characteristics.

	HIIT n	*MIC*E n	Total n (%)
Cancer Stage			
II	0	2	2 (29)
III	2	3	5 (71)
Treatment Modality			
Chemo (*n* = 7)	2	5	7 (100)
Radiation (*n* = 4)	1	3	4 (57)
Surgery (*n* = 7)	2	5	7 (100)
Cancer Treatment			
Unimodal	0	0	0
Bimodal	1	2	3 (43)
Multimodal	1	3	4 (57)
Sex			
Female	0	3	3 (43)
Male	2	2	4 (57)
Ethnicity			
Hispanic	0	0	0
Non-Hispanic	2	5	7 (100)
Race			
White	2	5	7 (100)
Marital Status			
Married	2	5	7 (100)
Not Married	0	0	0
Employment Status			
Employed	2	4	6 (86)
Unemployed	0	1	1 (14)
Household Income			
$40,000-$59,999	0	2	2 (29)
$60,000-$79,999	1	0	1 (14)
$80,000-$100,000	1	2	3 (43)
>$100, 000	0	1	1 (14)
	**HIIT** **Raw Data**	**MICE** **Median (1**^**st**^**, 3**^**rd**^ **Quartile)**	**Total** **Median (1**^**st**^**, 3**^**rd**^ **Quartile)**
Age (years)	39, 44.5	37 (34, 50)	39 (36, 50)
BMI (kg/m^2^)	24.5, 25.7	28.5 (23.8, 36.1)	27.4 (24.5, 29.7)

HIIT = High Intensity Interval Training (n = 2), due to n of 2 raw data provided for age and BMI; MICE = Moderate Intensity Continuous Exercise (n = 5).

### Feasibility and acceptability of the remotely supervised exercise intervention

On average, participants completed 88.6% (95% CI 79.4, 97.8; range: 63–100%) of the number of prescribed workouts throughout the 12-week intervention. Participants, in both groups, self-selected the mode of weight-bearing aerobic exercise. Most participants reported either walking or running as their mode of exercise. Among participants assigned to MICE, adherence was 95.6%. For HIIT, average adherence to the high-intensity intervals was 28.8%, and 51% during active recovery intervals. Table 2 displays adherence of participants to the high-intensity and active recovery intervals in the HIIT group. Low adherence to the exercise prescription was attributed to elevated heart rate beyond the prescribed heart rate training zones for the high-intensity and active recovery intervals.

**Table 2:** Percent of training intervals vonsistent with the prescribed heart rate zones among HIIT participants (*n = 2)*.

	Warm-up (%)	High-Intensity Interval (%)	Active Recovery Interval (%)
Interval 0	74	n/a	n/a
Interval 1	n/a	21	53
Interval 2	n/a	24	53
Interval 3	n/a	31	47
Interval 4	n/a	33	52
Interval 5	n/a	35	50

HIIT = High Intensity Interval Training.

Data from EOS assessments were used to evaluate acceptability of the intervention. Thematic analysis from EOS assessments related to the exit survey prompt ‘what participants liked about the exercise intervention’ coalesced around the themes of ‘personal accountability’, ‘motivation’, ‘improved quality of life’, ‘choice and control over workout intensity’, and ‘nudge’ to be active following the intervention. Table 3 provides detailed quotes related to each theme. All the participants noted in the EOS assessments their plan to continue exercise.

**Table 3:** Results from qualitative thematic analysis of open text responses.

Theme	Quote
Accountability	“I liked the time; it gave me a reason to go get exercise”; “getting it done”; “the responsibility of having to do it”
Motivation	“I liked the frequency/intensity”; “I like having a routine, something to push me”.
Improved quality of life	“Cleansing feeling, mental adjustment, time alone”; “long brisk walks and listen to audiobooks/podcasts while getting the dog out for a long walk”; “greatly helped with my stress levels”
Choice/Control over workout intensity (Autonomy)	HITT: “I had a hard time getting more higher intensity workouts in with my daily schedule “; “Mix up the 5 times a week into some moderate and some higher intensity because of my schedule”; “Mix up intensity”. MICE: “not hard enough for me”; “I might have increased the max heart rate just to push myself a bit”; “more intensity, and less overall time. My schedule is so packed that 60 minutes a day can be difficult—I would like to work out 30 minutes a day with more intensity for the 30 minutes”; “Heart rate parameters”; “I will exercise with a higher heart rate for varied lengths of time”; “I will exercise with a higher heart rate for varied lengths of time”; “…outdoor like to increase intensity a bit”; “Yes. Just a bit more intense.”
Technical issues	“Heart rate monitor was large on me”
‘Nudge’ to be active following the intervention	“Yes, I plan to exercise more. I plan to mix it up with different activities that I can fit into my schedule but still strive for the goal of 5x a week”; “yes, I am going to start training again for a half marathon in May”.

### Change in health outcomes from baseline to end-of-study

Table 4 provides detail by group regarding change in health outcomes evaluated during the assessment sessions at baseline and end-of-study.

**Table 4:** Change in health outcomes from baseline to end-of-study.

Heath Outcome	HIIT (n = 2) Mean (95%CI)	MICE (n = 5) Mean (95% CI)
Anthropometrics		
Weight (kg)	0.31(-0.60, 1.22)	-0.29 (-2.72, 2.14)
BMI (kg/m^2^)	0.11 (-0.25, 0.48)	-0.08(-0.90, 0.74)
Body Composition		
Fat-Free Mass (kg)	0.54 (-3.73, 4.81)	-0.63 (-2.71, 1.45)
Fat Mass(kg)	-0.23 (-5.35, 4.89)	0.34 (-1.99, 2.67)
% Body Fat	-0.50 (-8.12, 7.12)	0.40 (-1.76, 2.56)
% Fat-Free Mass	0.50 (-7.12, 8.12)	-0.40 (-2.56, 1.76)
Physical Function		
Handgrip Strength (kg)	0.75 (-8.78, 10.28)	2.25 (.35, 4.14)
Short Physical Performance Battery Score	0.00 (N/A)	0.80 (-.24, 1.84)
Walking Speed (seconds)	0.00 (N/A)	0.00 (N/A)
Standing Balance Score	0.00 (N/A)	0.00 (N/A)
Sit-to-Stand (seconds)	0.00 (N/A)	0.80 (-.24, 1.84)
PROMIS Physical Function Score	-3.90 (-53.45, 45.65)	3.2 (-5.83, 12.23)
Chemotherapy-induced Peripheral Neuropathy		
Neuropathy Total Symptom Score-6	.50 (-31.21, 32.20)	0.33 (-2.56, 3.23)
Utah Early Neuropathy Scale (Score)	-1.00 (-13.71, 11.71)	0.00 (-1.76, 1.76)
Physical Activity (MET-hr./wk.)		
Mild-Intensity Activity	2.8 (-24.7, 30.4)	2.5 (-3.0, 8.0)
Moderate-Intensity Activity	4.2 (-17.0, 25.3)	10.9 (-4.5, 26.3)
Strenuous-Intensity Activity	15.2 (0.3, 30.0)	-1.3 (-8.4, 5.8)

N/A = 95% confidence intervals could not be calculated because standard deviation is zero; HIIT = High Intensity Interval Training; MICE = Moderate Intensity Continuous Exercise.

### Anthropometrics & body composition

On average, participants experienced a 0.12 kg (95% CI -1.33, 1.09 kg)) reduction in body weight upon completion of the intervention. Mean change in body composition for all participants was as follows: -0.30 kg fat-free mass (95%CI -1.41, 0.81 kg), -0.14 percent fat-free mass (95%CI -1.27, 0.99%), 0.18 kg fat mass (95%CI -0.98, 1.34 kg), and 0.14 percent body fat (95%CI -0.99, 1.27%). Participants in HIIT experienced an increase in fat-free mass and percent fat free mass, along with a reduction in fat mass and body fat percentage. Participants in MICE experienced reductions in fat-free mass, percent fat-free mass, increases in fat mass and body fat percentage.

### Handgrip strength

Descriptive statistics indicate a change in handgrip strength for all participants from baseline to EOS was 1.82 kg (95%CI 0.71, 2.93 kg), however, change in handgrip strength did not meet or exceed established Minimal Clinically Important Difference thresholds.

### Short physical performance battery (SPPB)

We observed a mean change in SPPB score for all participants of 0.57 (95%CI -0.02, 1.15) with no change observed in HIIT and a minor increase in strength observed in MICE. No changes were observed in the HIIT group, with a modest increase in Sit-to-Stand time observed in the MICE group. Change in SPPB score did not meet or exceed established Minimal Clinically Important Difference thresholds.

### PROMIS physical function

Mean change in PROMIS Physical Function score was 1.17 (95%CI -4.19, 6.53) among all participants. Participants in HIIT experienced a decline in PROMIS physical function score, whereas participants in MICE experienced an increase.

### Neuropathy total symptom score-6 (NTSS-6)

Descriptive statistics indicate a change in NTSS-6 score for all participants was 0.38 (95%CI -1.39, 2.15), with participants in HIIT experiencing greater improvements in reported signs and symptoms of CIPN compared with MICE.

### Utah early neuropathy scale (UENS)

Change in UENS score for all participants was -0.29% (95%CI -1.31, 0.73)., with participants in HIIT experiencing greater improvements in reported signs and symptoms of CIPN compared with MICE.

### Modified Godin leisure time physical activity questionnaire (Godin)

Results from the modified Godin revealed increases in self-reported mild-, moderate-, and strenuous-intensity MET-hrs./wk. for all participants (mild: 2.6 MET-hrs./wk., 95%CI -0.29, 5.49; moderate: 9.0 MET-hrs./wk, 95%CI 1.07, 16.9; strenuous: 3.4 MET-hrs./wk, 95%CI -3.49, 10.3). Among participants in HIIT, increases were observed for MET-hrs./wk. of mild, moderate, and strenuous physical activity, with an increase in strenuous activity from baseline. For participants in MICE, MET-hrs./wk. of mild- and moderate-intensity exercise increased yet MET-hrs./wk. engaged in strenuous activity decreased.

## Discussion

Among the seven participants in our trial, we found that stage II-III CRC survivors’ post-resection and adjuvant therapy tolerated and expressed acceptability for an mHealth, home-based exercise intervention. We observed an 88.6% completion rate of prescribed workouts, 100% retention rate, no adverse events, and qualitative data indicating improved quality of life and positive feedback related to ease of use, accountability, motivation, and autonomy. Our exercise intervention included HIIT and MICE exercise prescriptions. The inclusion of HIIT addresses time, an important barrier to exercise engagement among cancer survivors [20,24]. Previous HIIT interventions among cancer survivors were carried out across the care continuum (e.g., before, during and after treatment) [51–57]. Our exercise intervention allowed for personal choice in type of aerobic exercise, and did not necessitate aerobic exercise equipment or access to a gym. Trends in our study related to feasibility, safety, and acceptability of HIIT are consistent with previous research among cancer survivors [30–33,53–56,58–60]. An important caveat is that only one portion of our definition of feasibility for HIIT was met. While our participants met our benchmark to prescribed exercise completion rates, ≥ 75% of prescribed workouts, participants were not adherent to the heart rate training zones within the HIIT exercise prescription. Qualitative data revealed that staying within the designated heart rate training zones was difficult; participants wanted to work out harder than their individualized exercise prescription. This information is important when considering heart rate training zones for future HIIT interventions in this patient population.

In addition to our feasibility results, findings related to safety support previous research using HIIT interventions [57]. Our high retention rate is consistent with a previously administered mHealth PA intervention among older individuals with cancer [61]. The home-based nature of our intervention, along with our discoveries related to ease of use and motivation aligns with reported preferences for mHealth exercise interventions among cancer survivors, such that cancer survivors prefer mHealth interventions that are easy to access and enhance personal motivation [62]. Previous research indicates that exercise adherence has a positive correlation with geographical ease of access to exercise support in cancer survivors [63]. Our qualitative data also indicated that participants planned to continue exercising after the intervention ended. Acceptability of our intervention may be attributed to the fact that our intervention implemented identified key elements for success in mHealth exercise interventions and preferences for mHealth interventions among cancer survivors, which includes: self-monitoring, a focus on increasing exercise engagement versus other outcomes, ease of access, and enhanced personal motivation [62,64]. Together, the descriptive findings from our study suggest that there is a place for mHealth HIIT delivered interventions as an aerobic exercise prescription for stage II-III CRC survivors’ post-resection and adjuvant therapy that warrants further investigation in a fully powered study.

In the present investigation, participant trends indicate a minor reduction in weight after 12 weeks, which has been shown in previous research from a 10-week mHealth delivered exercise intervention in post-treatment breast cancer survivors [65]. mHealth interventions may be utilized as a tool to help facilitate weight loss, which is important when considering the link between overweight/obesity status and cancer [66]. Among all participants we observed improving trends in physical function with exercise. Previous research that has utilized mHealth delivered exercise interventions among diverse populations [67–69], have used the SPPB to assess physical performance and observed significant improvements in individual components of SPPB and overall SPPB scores [68,69]. Improvements in human performance and physical function are important, as these factors are linked with improvements in survivorship, and survival in some cancer types [70].

Exercise patterns and increased engagement in CRC survivors have been used as a prognostic indicator of quality of life and improved survivorship [71]. We observed increases in exercise engagement as measured by the modified Godin questionnaire that aligns with previous mHealth exercise interventions delivered among breast cancer survivors [72]. Our descriptive findings warrant further investigation in a fully powered trial given previous studies suggest mHealth delivery is a viable choice to enable exercise engagement. mHealth delivered exercise interventions have exhibited small to moderate increases in exercise levels [73], and greater increases in daily step counts compared with an in-person, group exercise intervention [74].

We are the first, to our knowledge, to evaluate an mHealth, home-based delivered exercise intervention, including a HIIT prescription, among stage II-III CRC survivors’ post-resection and adjuvant therapy. We consider this a strength of our work. Other strengths include: a personalized exercise prescription based on measured peak heart rate, choice of exercise modality to complete the exercise prescription, a 4-week progression to goal exercise prescription, and inclusion of qualitative data to place results in context and further inform feasibility and acceptability of the intervention. Our trial is not without limitations. When measuring heart rate during exercise with the Polar device, there is a short delay in heart rate measurement output when changing exercise intensity. This likely contributed to some of the apparent non-adherence to measured heart rate within the prescribed training zones. Additionally, we had a homogenous and small sample within this pilot study. The small sample size is related to challenges with accrual secondary to the impact of the COVID-19 pandemic on in-person research, as our trial required in-person assessment sessions. Recruitment was on hold at the start of the pandemic and resumed approximately 3.5 months later; however, potential participants were still hesitant to attend assessment sessions in person. Our experience accruing and carrying out our trial during the beginning of the pandemic echoes what has been found in previous research, where clinical trials that undergo even short temporary suspension can lead to trial breakdown[75]. Upon resuming accrual, some of our in-person assessment procedures were modified, which negatively impacted accrual.For example, we had one prospective participant who wanted to complete a cardiopulmonary exercise test and declined participation since we could not carry out this testing at that time due to COVID-19. Future work is needed evaluating the efficacy of mHealth home-based exercise interventions among cancer survivors, and testing these interventions among a larger, heterogenous population of CRC survivors. More work is needed evaluating the feasibility of HIIT prescriptions in terms of adherence to heart rate training zones.

## Conclusion

Our findings indicate an mHealth, home-based delivered exercise intervention, including a HIIT prescription, among stage II-III CRC survivors’ post-resection and adjuvant therapy was tolerable and showed trends towards acceptability. Our qualitative data revealed that survivors wanted to work out harder/do more. Collectively, this work supports the utility of mHealth, home-based exercise interventions for cancer survivors, while also supporting the use of HIIT as a mode for aerobic exercise delivery in stage II-III CRC survivors post-resection and adjuvant therapy within their 5-year surveillance period.

## Supporting information

S1 Checklist CONSORT 2010 checklist of information to include when reporting a randomised trial*. (DOC)

S1 File (XLSX)

S2 File (PDF)
