# Supplementary material for: Feasibility and acceptability of an mHealth, home-based exercise intervention in colorectal cancer survivors: A pilot randomized controlled trial
Source: PLoS One. 2023 Jun 22;18(6):e0287152. doi: 10.1371/journal.pone.0287152 (PMC10286977; doi:10.1371/journal.pone.0287152)
Supplement: S2 File — (PDF) [file pone.0287152.s003.pdf]

IRB_00122238 **Created:** 4/19/2019 11:16 AM

PI: Adriana Coletta **Submitted:** 7/1/2019

Title: Home-based high-intensity interval training to improve colorectal cancer survivorship: feasibility and relationship with novel surrogate biomarkers of colorectal cancer recurrence

IRB_00122238
1. Contacts and Title

1. Study Introduction

1. **Responsible Investigator:**
   Adriana Coletta

Email	Training	Col Date
adriana.coletta@hci.utah.edu	8/5/2021 SMCG	8/24/2022

   a. **Position of the Investigator:**
   - (●) **Faculty or** Non-Academic Equivalent
   - ( ) Student
   - ( ) Staff
   - ( ) Resident/Fellow
   - ( ) Other

2. **Contact Persons for the Responsible Investigator:**

Name	Email	Training
Lindsey Martineau		5/19/2020 MCG
Kimberly Norman		9/3/2020 MCG

3. **Guests of the Responsible Investigator:**

Last Name	First Name	E-Mail
Berry	Therese	
Ma	Debra	
Mathis	Elizabeth	
Sama	Neetha Reddy	

4. **What type of application is being submitted?**
   New Study Application (or Amendment/Continuing Review)

5. **Title Of Study:**
   Home-based high-intensity interval training to improve colorectal cancer survivorship: feasibility and relationship with novel surrogate biomarkers of colorectal cancer recurrence

6. **Study Purposes and Objectives:**
   This 12-week trial will randomize colorectal cancer (CRC) survivors post resection and adjuvant therapy into one of two groups for a home-based exercise intervention: high-intensity interval training (HIIT) or moderate-intensity continuous exercise (MICE). Assessments will be conducted at baseline and 12

weeks (end-of-study).

Primary objective and endpoint:

1. To demonstrate the feasibility of home-based HIIT among CRC survivors. Feasibility will be assessed by HIIT adherence, which is defined at the participant level as completing ≥70% of workouts consistent with the exercise prescription. The intervention will be considered feasible if ≥75% of participants meet or exceed the 70% criterion.

Secondary objectives and endpoints:

1. To generate evidence on the magnitude of the effect sizes and outcome variability of physical outcomes linked with CRC survival and quality of life (body composition, physical function, fitness, and chemotherapy-induced peripheral neuropathy (CIPN)) for home-based HIIT and home-based MICE. Outcomes to be measured at baseline and 12 weeks include body composition, physical function, cardiorespiratory fitness, and CIPN.

2. To explore the relationship between changes in exercise behavior and changes in novel surrogate biomarkers of CRC recurrence. Plasma will be collected at baseline and 12 weeks to measure biomarkers of CRC recurrence.

This pilot study will provide preliminary data for a larger trial that will be proposed in an R01 grant.

7. **Is this a multi-site study, where more than one site needs IRB approval?**
   ◯ Yes ● **No**

8. **Background and Introduction:**

Rationale and Background:

Hypothesis: We hypothesize that home-based HIIT will be feasible among stage II-III CRC survivors post resection and adjuvant therapy.

Colorectal cancer (CRC) is the fourth most common cancer in the USA, affecting about 1.3 million men and women[15]. The estimated direct medical cost of CRC care is $14 billion per year[16]. Among CRC survivors, epidemiological evidence supports that regular exercise promotes a 50% reduction in CRC recurrence at fairly high doses (≥18 MET [metabolic equivalent] hours/week), and promotes a 39% reduction in CRC-specific mortality and 42% reduction in total mortality[17-22]. Furthermore exercise augments improvements in physical outcomes associated with CRC survival and quality of life, such as body composition, physical function, fitness, and chemotherapy-induced peripheral neuropathy (CIPN)[23-31]. Yet most CRC survivors do not engage in regular exercise[32]. **One of the major research priorities in the field is to test the relationship between exercise and survival outcomes in a randomized controlled trial, and thereby identify an optimal, yet feasible and acceptable, exercise prescription to improve disease-free and overall survival.**

Of the few randomized controlled exercise trials conducted among stages I-IV CRC survivors post resection and adjuvant therapy, moderate-intensity continuous exercise (MICE) has been tested and is considered effective in facilitating improvements in body composition[33,34], physical function[35-38], and fitness[34,36,39]. Aerobic exercise is also associated with a reduction in signs and symptoms of CIPN among CRC survivors post resection and adjuvant therapy[40]. Epidemiological evidence demonstrates a 50% reduction in CRC recurrence with a fairly high dose of exercise (≥18 MET [metabolic equivalent] hours/week)[17], which may be difficult to attain with MICE alone due to the time commitment required to achieve this higher dose (at least 60 minutes per day, 5 days per week). A common reported barrier to exercise among CRC survivors is lack of time[41,42]. High-intensity interval training (HIIT) requires less time than MICE while providing a higher dose (i.e.- increased MET hours) of exercise due to the increase in intensity. Thus a potential advantage of HIIT in this population is that it may facilitate increased participation in exercise due to a reduction in total weekly time commitment. In other clinical populations, HIIT revealed equivalent or greater improvements in physical function, body composition, and fitness compared to MICE[43-53]. HIIT has been compared to MICE in two studies with male and female CRC survivors in a supervised setting[54,55]. These studies differed in length of intervention; one study was 4 weeks[55] in length and the other 8 weeks[54]. Collectively, both trials demonstrated feasibility (≥96.7% study completion rate and ≥99.7% exercise prescription compliance) and safety (no severe adverse events reported) of supervised HIIT in stage I-IV CRC survivors at least one-month post-treatment. Further, significant improvements were observed only in the HIIT group for body composition and fitness in both trials (4-week trial: -0.74 kg fat mass, +3.5 mL/kg/min; 8-week trial: -0.7 kg fat mass, +3.3 mL/kg/min). Thus, HIIT may serve as a superior alternative to MICE among both male and female CRC survivors to improve body composition and fitness. However, **there are no data on the feasibility of HIIT under free-living conditions, such as an unsupervised home-based setting**, which may be more likely to be sustained, as most cancer survivors identify a preference for exercise they can do on their own[56]. Home-based methods have been shown to increase exercise among CRC survivors acutely and long-term[23,57], thus warranting further investigation into home-based HIIT within this population.

In addition to identifying the most effective and sustainable exercise prescription to improve CRC survival outcomes, further research is needed understanding the link between exercise and reduced CRC recurrence. Exercise trials with survival endpoints are ideal, but take many years to complete. Surrogate biomarkers of disease recurrence can speed research on this topic to provide answers about intervention effectiveness. Currently, **the effect of exercise on surrogate biomarkers of CRC recurrence is unknown.** To date, one trial, currently in progress, will assess the relationship between a mix of supervised and home-based moderate- to vigorous-intensity exercise, providing a dose of 10-12 MET-hours/week, and CEA (carcinoembryonic antigen)[58]. CEA is a surrogate biomarker that can be used for early diagnosis of recurrent CRC when used serially. Yet when comparing CEA to circulating tumor DNA (ctDNA), evidence favors ctDNA as a more accurate marker to predict recurrent disease postoperatively[59]. Recent research demonstrates 50-60% sensitivity and >95% specificity of ctDNA for predicting recurrence[60-65]. No research to date has assessed changes in ctDNA levels in relation to exercise behavior or differences in exercise prescription. Assessment of ctDNA in addition to CEA is novel to understand the relationship between exercise and reduced CRC recurrence.

The scientific premise of this trial rests on epidemiologic data indicating a relationship between exercise after CRC diagnosis and CRC recurrence and disease-free survival; the benefits of exercise for survivor physical functioning, body composition, fitness, and reductions in signs and symptoms of CIPN; the effectiveness of HIIT in other clinical populations; and the accuracy of ctDNA as a surrogate biomarker of CRC recurrence. The proposed research will improve scientific knowledge and contribute to the exercise and oncology fields by determining the feasibility of a potentially advantageous

exercise intervention (home-based HIIT) to reduce CRC recurrence and improve survival, while also exploring the relationship between exercise and novel surrogate biomarkers of CRC recurrence. The data from this project will inform a larger, potentially practice-changing, trial that will determine whether exercise dose (via manipulation in intensity and volume) impacts changes in biomarkers facilitating prevention of CRC recurrence and improvements in disease-free survival, and affects exercise adherence for sustained lifestyle change. An effective and acceptable exercise prescription may result in reduction of CRC recurrence rates and health care costs over time.

Please see attached for references.

IRB_00122238 **Created:** 4/19/2019 11:16 AM

PI: Adriana Coletta **Submitted:** 7/1/2019

Title: Home-based high-intensity interval training to improve colorectal cancer survivorship: feasibility and relationship with novel surrogate biomarkers of colorectal cancer recurrence

IRB_00122238
2. Study Location and Sponsors

2. Study Location and Sponsors

1. **Add all locations applying for approval of research via the University of Utah IRB or Human Research Protection Program (HRPP).**

   Click the appropriate button(s) below to add locations:

	Site Name	Investigators Name	Covered Entity	Sub Sites
View	University of Utah	Adriana Coletta	No<br>Yes	

2. **Will a Central IRB (CIRB) or Single IRB (SIRB) model be used for review of this study for the sites listed in this application?**
   ◯ Yes ● **No**

3. **Indicate the source(s) of funding obtained or applied for to support this study.**

	Sponsor	Sponsor Type	Sponsor Contact Information	Prime Sponsor	Prime Sponsor Type	OrgID
View	HUNTSMAN CANCER INSTITUTE	UofU	Adriana Coletta's Research Start-up Funds and Supportive Oncology and Survivorship Center Pilot Project Award			215

4. **Does this study have functions assigned to a Contract Research Organization (CRO)?**
   ◯ Yes ● **No**

5. **Does this study involve use of the Utah Resource for Genetic and Epidemiologic Research (RGE)?**
   Examples: Utah Population Database (UPDB), Utah Cancer Registry (UCR), All Payers Claims Database (APCD), etc.
   ◯ Yes ● **No**

IRB_00122238 **Created:** 4/19/2019 11:16 AM

IRB_00122238
View/Edit

PI: Adriana Coletta **Submitted:** 7/1/2019

Title: Home-based high-intensity interval training to improve colorectal cancer survivorship: feasibility and relationship with novel surrogate biomarkers of colorectal cancer recurrence

Addition of a Site

1. **Site Name:**
   University of Utah

2. **Site Principal Investigator**
   ☑ **Mark if Same as Responsible Investigator (syncs with investigator on the first page)**
   Adriana Coletta

Email	Training	CoI Date
adriana.coletta@hci.utah.edu	8/5/2021 SMCG	8/24/2022

   a. **Position of the Site Principal Investigator**
   Faculty or Non-Academic Equivalent

   b. **Will the Site PI consent participants?** ◉ **Yes** ○ No

3. **Site Contact Persons, if different from the Site PI:**
   ☑ **Mark if Same as Contacts for Responsible Investigator (syncs with contacts on the first page)**

Name	Email	Training
Lindsey Martineau		5/19/2020 MCG
Kimberly Norman		9/3/2020 MCG

4. **Site Staff and Sub-Investigators**

Name	Email	Training	Obtaining Consent	CoI Date
Kristin Barber		10/12/2021 MCG	☐	9/21/2022
Emily Dunston		8/29/2020 SMG	☐	7/20/2022
Ignacio Garrido-Laguna		9/28/2022 MCG	☐	10/6/2022
Glynn Gilcrease		6/24/2020 MCG	☐	10/10/2022
Benjamin Haaland		9/1/2022 MCG	☐	10/6/2022
Paula Hobson		9/29/2020 MCG	☐	6/28/2022
Allie Memmott		4/9/2020 MCG	☑	10/20/2022

Name	Email	Training	Obtaining Consent	CoI Date
Ann Marie Moraitis		1/29/2022 MG	☐	1/26/2022
Kimberly Norman		9/3/2020 MCG	☑	5/18/2022
J. Robinson Singleton		3/23/2020 MCG	☐	6/29/2022
Benjamin Solomon		4/6/2022 MCG	☐	9/21/2022

5. **Site Guests:**

Name	Email	Training

There are no items to display

6. **Select HIPAA coverage for this study:**
Study procedures will be conducted within a HIPAA Covered Entity at this site (HIPAA Privacy Rule applies)
Study procedures will be conducted outside a HIPAA Covered Entity at this site (HIPAA Privacy Rule does not apply)

7. **Select the study procedures that will be conducted at this site:**
Recruitment
Consent/Enrollment
Research observation/intervention with participants
Data collection
Data analysis

Do you have an enrollment goal or anticipated enrollment number for this site?

● **Yes**
○ No

Enrollment Number:
up to 50

8. **Select the University of Utah department responsible for this research:**
HUNTSMAN CANCER INSTITUTE

9. **Add any additional sites that are part of this performance group**
There are no items to display

IRB_00122238 **Created:** 4/19/2019 11:16 AM

PI: Adriana Coletta **Submitted:** 7/1/2019

Title: Home-based high-intensity interval training to improve colorectal cancer survivorship: feasibility and relationship with novel surrogate biomarkers of colorectal cancer recurrence

IRB_00122238
IRB Smart Form

Sponsor Information

a. **Are you receiving award or contract management for the sponsored funds through the University of Utah Office of Sponsored Projects?**

○ Yes ● **No**

If no, indicate how the funds are being received:
Department

Sponsor:
HUNTSMAN CANCER INSTITUTE

Previously, the following data was entered on your IRB application:

Sponsor Contact Information:
Adriana Coletta's Research Start-up Funds and Supportive Oncology and Survivorship Center Pilot Project Award

IRB_00122238 **Created:** 4/19/2019 11:16 AM

PI: Adriana Coletta **Submitted:** 7/1/2019

Title: Home-based high-intensity interval training to improve colorectal cancer survivorship: feasibility and relationship with novel surrogate biomarkers of colorectal cancer recurrence

IRB_00122238
3. Participants

3. Participants

1. **Ages of Participants:**

   18 and older (Consent form needed)

2. **Specific age range of participants (e.g., 7-12 years old, 60+, etc.):**
   19-75

3. **Indicate any vulnerable participant groups (other than children) included:**

   None

   If "Other", please specify:

   If "None" and no children are involved, answer the following question.
   Has the participant selection process overprotected potential subjects who are considered vulnerable so that they are denied opportunities to participate in research?
   ○ Yes ● **No**

4. **Number of participants to be included and/or enrolled in this entire study, across all study locations:** up to 50

   At Utah prior to October 2019: up to 50

5. **Characteristics of Participants/Inclusion Criteria:**

   1. Patients who are no more than five years post resection and/or adjuvant therapy for stage II-III Colorectal cancer (CRC).
   2. Age 19-75 years old.
   3. No known cardiovascular disease, with the exception of hypertension that is under control via medication, metabolic or renal disease, and signs/symptoms suggestive of cardiovascular, metabolic, or renal disease
   4. Must be able to read, speak and understand English
   5. Willing to complete two assessment sessions (at baseline and 12 weeks).
   6. Willing to engage in moderate- or vigorous-intensity aerobic exercise at home and use mobile health technology to track exercise adherence to the exercise prescription.
   7. Able to provide informed consent and willing to sign an approved consent form that conforms to federal and institutional guidelines.
   8. Have regular access to a smart phone and willing to download a free application for device tracking

6. **Participant Exclusion Criteria:**

   1. Functional limitations requiring a walker, scooter, or wheelchair.
   2. Clinically evident recurrent disease.
   3. Resting blood pressure ≥140/90 at the time of baseline testing.
   4. No access to smart phone and/or not willing download the device app

7. **Is a substantial percentage of the participant population anticipated to be non-English speaking?**

   ○ Yes ● **No**

IRB_00122238 **Created:** 4/19/2019 11:16 AM

PI: Adriana Coletta **Submitted:** 7/1/2019

Title: Home-based high-intensity interval training to improve colorectal cancer survivorship: feasibility and relationship with novel surrogate biomarkers of colorectal cancer recurrence

4. Study Information

1. **Design of Study (select all that apply):**

   ☐ **Non-Experimental and/or Descriptive Research Design:**

   There are no items to display

   ☑ **Experimental and/or Interventional Research Design:**

   Prospective Biomedical Intervention or Experiment
   Randomized Trial

   ☐ **Development of a research resource (repositories, databases, etc.)**

   There are no items to display

   ☐ **Other**

2. **Does your study involve the use of any placebo?**
   ○ Yes ● **No**

3. **Length of entire study, from initiation through closeout:**
   2 years

4. **How will participants be recruited or identified for inclusion in the study?**

   a. **Select all methods that will be used:**

   In-person contact (e.g., patients, students, etc.)

   Referrals

   Written or electronic record review

   Written advertising (flyers, brochures, website postings, newspaper ads, etc.)

   From a database or participant pool for which participants have given prior permission to be contacted for research studies

   Other

   Human Subjects Recruitment Tool (HSRT)

   b. **Describe the recruitment/participant identification process in detail (e.g. who will review charts or records, who can refer participants to the study, where will flyers be posted, how often will recruitment letters be sent, when will follow-up phone calls be made, etc.):**

   1) In person contacts: identified from clinic lists in the electronic health record. Study coordinator will approach in clinic at Huntsman Cancer Institute and/or University Hospital during a standard of care clinic visit.

   2) Referred by physicians, and their support staff, at Huntsman Cancer Institute, University of Utah Health, and Intermountain Healthcare. This would be done by the providers presenting potential participants with study flyer for more information. Distribution of the in-person flyers may be as frequent as clinic visits.

   3) Electronic Health Record Review: Identify possible participants, then provide flyer as described next. Wavier requested.

3) Flyers: distributed through mail, email, and/or MyChart. The in-person flyers may be distributed as frequent as clinic visits. Likely coinciding with their standard of care follow-up.

4) Database review from the HCI Research Subject Registry (RSR) database by study coordinator. This would only be for other studies that have participants who have consented to be contacted with future research opportunities.

5) Recruitment Letter: potential participants identified from record review (electronic health or research subject registry) may be sent this letter with study flyer attached.

6) HSRT: Another method that may be used for recruitment with support from the Data Science Services Office at the UofU, is use of the web-based Human Subjects Recruitment Tool (HSRT). Using structured and unstructured data from the electronic health record, the HSRT will be used to help screen for potentially eligible patients. Data from HSRT will become unavailable the day the IRB expires or when the research team notifies the EDW that the recruitment period closes.

5. **How will consent be obtained?**

Informed Consent Process (with or without a document)

6. **Describe all the procedures chronologically, from screening/enrollment through study closeout, which will be completed in the research project.**

Screening, recruitment and consent process will occur, as outlined in this ERICA application. Involvement in this study will involve a medical release form. Mainly for verification of eligibility criteria, verification of cancer diagnosis and treatment, and access to possible CEA lab result reports.

Study Coordinator will schedule with participants the baseline visit at the SKAGGS Wellness Center, which is estimated to take up to 3 hours. If participant provided phone or email consent, study coordinator will have the participant sign a clean informed consent form in-person at the baseline visit to ensure that consent is fully given. Once done, participant will partake in the baseline session activities.

It is estimated that participants may be involved in this study for up to 16 weeks, but anticipated average is 12 weeks. This variability may be due to scheduling conflicts for SKAGGS, delay in medical records for eligibility criteria, and also the possibility of participant travel or other needs to take a temporary break from the exercise prescription.

Randomization will occur in a 1:1 ratio by gender stratification, with available calculation software in the REDcap system. All questionnaires/surveys (demographic, PROMIS, CIPN, Godin, exit interview) will be given to participant through the HCI REDcap incidence, which will link to the Research Subject Registry (RSR) database at HCI. Up to 50 patients may be enrolled in this study with the goal of obtaining 30 evaluable participants. The purpose of this parameter is to account for potential withdrawals after phone consent, withdrawals for other reasons as indicated in the protocol, or dropouts.

Intervention Procedures

Equipment and Exercise Protocol Familiarization Session- Following completion of baseline testing procedures, participants randomized to high-intensity interval training (HIIT) or moderate-intensity continuous aerobic exercise (MICE) will complete an Equipment and Exercise Protocol Familiarization Session pertaining to the fitness tracking device and exercise prescription within the exercise group they were randomized to. Participants will be provided with a Polar® A370 fitness tracking device and Polar® heart rate (HR) sensor (Polar Electro Inc., Lake Success, NY) to monitor HR during exercise. Study personnel will help participants set up the Polar fitness tracking and HR sensor devices and demonstrate how to use them during exercise. Upon completion of the setup process, participants will complete their first workout with the device, supervised by study personnel, on a treadmill. A study binder and follow-up email including written instructions of the information provided during the Equipment and Exercise Protocol Familiarization Session will be provided upon completion of the session.

A draft of the study binder, participant handbook outline is attached in Other Documents. Each binder will be individualized for the participants based on which exercise program they will follow. Study coorindator contact information will also be provided in this binder for any research related questions.

High-Intensity Interval Training (HIIT)- The goal HIIT regimen is to complete four, 50-minute HIIT workouts per week. The goal HIIT exercise prescription is as follows: 10-minute warm-up at 50-75% peak HR followed by five, 4-minute intervals at 85-90% peak HR with 4 minutes of active recovery at 50-75% peak HR following each 4-minute interval. The goal exercise prescription yields a weekly MET value within a range of approximately ≥18 MET hours/week. This prescription will include a progression in time and intensity to the goal HIIT prescription - Please refer to Table 1 below. Note total time estimates are per session, 4days/week.

Table 1: Progression in Time & Intensity to Goal HIIT Prescription		

Time-point	Time	Intensity
Week 1<br>(4 days/week)	10-minute warm-up<br>**2** × 4-minute intervals<br>*4-minute recovery interval following each high intensity interval	50-70% peak HR<br>**75-80% peak HR**<br>*recovery at 50-70% peak HR
	Week 1 Total Time: 26 minutes	
Week 2<br>(4 days/week)	10-minute warm-up<br>**3** × 4-minute intervals<br>*4-minute recovery interval following each high intensity interval	50-70% peak HR<br>**80-85% peak HR**<br>*recovery at 50-75% peak HR
	Week 2 Total Time: 34 minutes	
Week 3<br>(4 days/week)	10-minute warm-up<br>**4** × 4-minute intervals<br>*4-minute recovery interval following each high intensity interval	50-70% peak HR<br>**85-90% peak HR**<br>*recovery at 50-75% peak HR
	Week 3 Total Time: 42 minutes	
Weeks 4-12<br>(4 days/week)<br>GOAL	10-minute warm-up<br>**5** × 4-minute intervals<br>*4-minute recovery interval following each high intensity interval	50-70% peak HR<br>**85-90% peak HR**<br>*recovery at 50-75% peak HR
	Weeks 4-12 Total Time: 50 minutes	

Moderate-Intensity Continuous Aerobic Exercise (MICE)-The goal MICE prescription is as follows: five, 60-minute MICE workouts per week, with each workout completed at 50-70% peak HR. This exercise prescription will yield a MET value of approximately ≥ 18 MET-hours/week. This prescription will include the following progression in time: 30 minutes × 5 days in week 1, 40 minutes × 5 days in week 2, 50 minutes × 5 days in week 3, 60 minutes × 5 days in weeks 4-12 (goal).

Mode of Exercise- Participants will be able to select the mode of weight-bearing aerobic exercise (i.e. walking, jogging, elliptical, calisthenic exercises). Only weight-bearing activity may be performed since the goal HR zones are based on peak HR measured during weight-bearing exercise (i.e. cardiopulmonary exercise test conducted on a treadmill).

Monitoring Exercise Compliance- Study personnel will monitor participant adherence to HIIT and MICE (i.e. intensity: HR tracking throughout each workout; volume: date/time workouts are completed and duration of each workout) weekly with use of the Polar Flow for Coach application (see Feasibility Assessment section below for details re: Polar Flow for Coach application). At the beginning of each week, study personnel will call participants who did not complete all of their workouts as prescribed in the previous week to check in on progress and identify any challenges participants may be facing in adhering to the protocol. Motivational interviewing and problem-solving techniques will be applied as appropriate to help non-adherent participants get back on track with compliance to the study protocol.

Assessment Procedures

Assessment Sessions will be held at baseline and at 12 weeks (end of study) and will be held at the Skaggs Wellness Center and Health Professionals Education Building (HPEB) on the University of Utah campus.

Feasibility Assessment- Feasibility of HIIT will be assessed at 12 weeks. The intervention will be considered feasible if ≥75% of participants adhered to the HIIT exercise prescription. Adherence to the intervention is defined as completing ≥70% of the workouts (or 34 out of the 48 prescribed workouts) in a manner that is consistent with the exercise prescription. Consistency with the exercise prescription is defined as maintaining heart rate (HR) within all prescribed zones or +/-5 beats per minute outside of each zone throughout the workout. Adherence to the exercise prescription will be assessed using the Polar A370 fitness tracking device, Polar HR sensor, Polar Flow and Polar Flow for Coach applications (Polar Electro Inc., Lake Success, NY). The Polar A370 device enables participants to monitor and record HR during each workout. HR will be measured from the chest, as opposed to the wrist, with the Polar HR sensor chest strap. The Polar HR sensor is compatible with the Polar A370 device. In the event a participant forgets to use the chest strap for a workout, HR will still be recorded from the wrist sensor on the A370 device; however, use of the chest strap will be encouraged as it provides a more accurate measure. The A370 device synchronizes with the Polar Flow application on any smartphone device or the Polar Flow website on a computer. The Polar Flow application coordinates with the Polar Flow for Coach application. Study personnel will use the Polar Flow for Coach application to monitor each participant's exercise activity (i.e. HR throughout the exercise session, duration of exercise session, date exercise performed). When each participant sets up his/her Polar A370 device with study personnel during the Equipment and Exercise Protocol Familiarization Session (see above for details of the session), the participant will enable study personnel to have access to his/her Polar Flow account.

Demographic Questionnaire-only at the baseline visit, a survey about ethnicity/race, smoking habits, marital status, employment and other generic questions.

Blood Draw- A member of the study team will conduct each blood draw. Each participant's study period will align as closely as possible with his/her standard of care (SOC) quarterly clinic schedule in order to align CEA measurements with the study period.

Carcinoembryonic Antigen (CEA) Analysis - As part of SOC management of CRC survivors, CEA is measured quarterly and analyzed within the hospital's diagnostics laboratory. The results of CEA analysis will be available in the patient's medical record. Study personnel will attempt to abstract these lab values from the medical record and include them in the study database. If for some reason that enrollment does not coincide with SOC lab results for CRC, the study team may collect an additional blood sample for CEA analysis at ARUP, paid for through study funds. The collection would occur at baseline session and at end of study session. If the CEA lab value provided in the medical record is not within 4 weeks of the study session, the additional sample draw will occur for research use. This option is available to ensure data integrity for this study consideration, but data abstraction is the preferred method.

Circulating tumor DNA (ctDNA) Analysis - After blood collection, the sample for ctDNA analysis will be transferred to the Biorepository and Molecular Pathology (BMP) shared resource at HCI. Upon arrival blood samples will be processed and stored at -80° C until ready for analysis. ctDNA will be quantified to assess for the methylation of the following genes linked with CRC: WNT inhibitory factor 1 (WIF1) and neuropeptide Y (NPY). Methylation of ctDNA will be assessed as opposed to tumor-specific mutations because information regarding tumor specific mutations may not be available for all participants. Recent research supports the use of methylated ctDNA as a surrogate marker of CRC in place of tumor-specific markers by ddPCR[1].

Body Composition- Body composition will be measured with air displacement plethysmograph via BOD POD Gold Standard 2007A (COSMED USA, Concord CA) following standard procedures.

Physical Function- Physical function assessment consists of two components, muscular strength and physical performance.

Handgrip Strength- The handgrip strength test will be used as the marker of muscular strength.

Short Physical Performance Battery- The short physical performance battery will be used to assess physical function and performance. This method consists of three components to assess physical function and performance: walking speed, balance and sit-to-stand performance[3,4].

1. *Walking Speed*- Participants will be instructed to walk four meters, at 0% incline, as quickly as possible[3].
2. *Standing Balance*- Participants will be instructed to stand up with their feet in three different positions (side-by-side, tandem and semi-tandem) for 10 seconds each position[3].
3. *Sit-to-Stand Performance*- Participants will be instructed to rise from a chair for five times consecutively[3].

PROMIS Physical Function- The PROMIS Physical Function Short Form 6b will complement the objective measures of physical function. The questionnaire consists of six items requiring a response on a scale from "without any

difficulty” to “unable to do” for questions 1-4 and “not at all” to “cannot do” for questions 5 and 6. This questionnaire has an average reliability coefficient of about 0.95[5].

Cardiopulmonary Exercise Test- The cardiopulmonary exercise test will follow the modified Balke protocol[6]. The Balke protocol is being used as opposed to the most widely used Bruce protocol because it is considered the more appropriate method for clinical populations and untrained individuals[6-8]. Prior to testing, the gas analyzer will be calibrated against known concentrations and the flowmeter will be calibrated following standard procedures. The ParvoMedics TrueMax 2400 Metabolic Measurement System (Parvomedics Inc, Sandy, UT) will be used to measure oxygen consumption. A 12-lead electrocardiogram (ECG) will be included to measure HR at peak aerobic capacity and the tracing will be monitored for safety purposes. Prior to initiation of the modified Balke protocol, a 2-minute warm-up will be completed. Rating of perceived exertion, HR, blood pressure, and ECG will be collected at the end of every stage. The participant will continue the test until he/she feels unable to continue; the measurement at that point will be considered the peak aerobic capacity and HR. All participants will complete at least a 2-minute walking cool down. Peak HR measured during the baseline test will dictate exercise intensity for the intervention.

Chemotherapy-Induced Peripheral Neuropathy (CIPN)- The Neuropathy Total Symptom Score-6 (NTSS-6) and the Utah Early Neuropathy Scale (UENS) will be used to assess CIPN. The NTSS-6 is a 15-item questionnaire inquiring about whether or not (yes or no) an individual is experiencing the specified feeling in his/her legs and feet. The NTSS-6 has an internal consistency of $\alpha > 0.7$ and an intra-class correlation coefficient of > 0.9[9]. The UENS is a clinical examination scale focused on small fiber sensory function, and is more specific for small fiber sensory neuropathy than the Michigan Diabetes Neuropathy Scale or the Lower Limb portion of the Neuropathy Impairment Score (NIS-LL)[10]. The interrater reliability of UENS is 94%[10] and its diagnostic efficiency as measured by ROC area under the curve is > 0.9.

Godin Leisure Time Physical Activity Questionnaire- The Godin Leisure Time Physical Activity Questionnaire will be administered as another method (in addition to objectively measured activity data from the Polar fitness tracking device) for measuring change in exercise behavior. This questionnaire will ask participants to identify weekly frequency of participation in strenuous, moderate, and mild exercise. This questionnaire has a reliability coefficient of 0.81, along with significant correlations with cardiopulmonary fitness[11].

Exit Interview- All participants will complete a survey at the end of study that consists of open-ended questions inquiring about the completed exercise intervention. These data will be considered during the planning stages of the future trial that will compare the efficacy of the tested exercise prescriptions on biomarkers and physical outcomes linked with CRC survival.

The End of Study visit will occur approximately 12 weeks after the baseline visit. This is estimated to take at most 2 hours of participant time. The health tracking devices will be returned to the study team, repeat of some of the physical tests and questionnaires, completing with the exit interview survey.

Data Analysis will occur as outlined in this application.

Pandemic Procedures

When COVID-19 pandemic research restrictions will not allow us to run a baseline cardiopulmonary exercise test, an estimation equation for peak HR may be used to dictate exercise intensity for the intervention. End-of-study cardiopulmonary exercise test may be omitted if COIVD-19 pandemic research restrictions will not allow. Additionally, CEA measurement, if not readily available in the medical record per time window outline in our protocol, may be omitted if COVID-19 pandemic research restrictions will not allow.

Participants will still engage in their assigned exercise intervention for 12-weeks. While participation in the trial may last up to 16 weeks, actual participation may be longer if we are unable to carry out end-of-study assessments in a timely fashion if COVID-19 pandemic research restrictions will not allow.

7. **Are all procedures for research purposes only (non-standard or non-standard of care procedures)?**
 ○ Yes ● **No**

 If no, list the procedures that are performed for research purposes only (non-standard or non-standard of care procedures):

 If possible, the CEA report will be collected from medical records at standard of care clinical visits.

8. **Is there a safety monitoring plan for this study?**
 ● **Yes** ○ No

9. **Provide a summary of the statistical methods, data analysis, or data interpretation planned for this study. Factors for determining the proposed sample size (e.g., power) should be stated.**
Statistical Analysis

Patient characteristics will be summarized overall and separately within each treatment arm as number (percent) or mean (standard deviation), as appropriate, and tested via chi-squared or t-tests.

In addition to the feasibility benchmark of at least 75% of participants completing at least 70% of workouts, adherence rates will be estimated via 95% confidence intervals (CI)[12] across the following workout completion percentage targets: 60%, 70%, 80%, and 90%.

For the quantitative outcomes (changes in body composition, physical function, fitness, CIPN, ctDNA, and CEA), the means, including 95%CIs, will be summarized separately for each arm, and the mean difference between groups will be summarized along with 95% CIs. The mean differences between groups will be compared via two-sample t-tests. Additionally, standard deviations of the quantitative outcomes will be estimated in the context of linear models adjusting for treatment arm. Box-Cox transformations may be examined if responses show evidence of non-bell-shaped distribution[13]. Relationships between changes in exercise behaviors and changes in ctDNA and CEA will be examined via Pearson's or Spearman's correlation coefficients depending on graphical assessment of relationship shape, along with 95% confidence intervals.

Missing data will be handled via multiple imputation, which ensures results are valid under missingness-at-random (data values and missingness status are independent given the observed data), the most general data-centric missing data assumption. Estimates, such as mean difference-in-differences and log-odds ratios, and their corresponding standard errors will be pooled across multiple imputations[14].

Power and Sample Size

The primary aim of this study is to demonstrate the feasibility of home-based HIIT among CRC survivors. Given the small sample size (n=30), the study will only be well-powered to detect very large differences between the treatment arms. Assuming a study completion rate of at least 80%, 15 participants randomized per arm will ensure at least 80% power for detecting mean differences-in-differences between the treatment arms of 1.15 standard deviations (of the individual level differences) and rate differences of 0.55.

IRB_00122238 **Created:** 4/19/2019 11:16 AM

PI: Adriana Coletta **Submitted:** 7/1/2019

Title: Home-based high-intensity interval training to improve colorectal cancer survivorship: feasibility and relationship with novel surrogate biomarkers of colorectal cancer recurrence

IRB_00122238
- Consent Process

Consent Process

1. **The following investigators and internal staff will obtain consent (as indicated on the Study Location and Sponsors Page):**

Adriana Coletta	**(PI)** University of Utah
Allie Memmott	University of Utah
Kimberly Norman	University of Utah

List by name, role, and affiliation any others who will obtain consent (e.g. Dr. John Smith, Co-Investigator, etc.).

2. **Describe the location(s) where consent will be obtained.**
Over phone: study staff will be in restricted access office space

Clinic areas at University Utah Health, Huntsman Cancer Institute, Intermountain Healthcare: participants may be handed a flyer, but asked to email or call study team for further involvement and/or information.

Clinic areas at University of Utah Health and Huntsman Cancer Institute only: Completion of consent can be done in various clinic or research reserved locations, generally at standard of care visits.

SKAGGS Wellness Center: space will be reserved for study team and participant to complete procedures, including consent.

3. **Describe the consent process(es), including the timing of consent. Describe whether there is a waiting period between the consent process and obtaining consent from the participant (i.e., any time between informing participants and actually obtaining consent).**
Participant will likely receive a flyer (via clinical staff, support staff, mail, email or MyChart), and may consider for as long as they need to reach out for more information. If flyer is sent with recruitment letter, study team will follow up with potential participant 3-7 days from sending.

Once a potential participant contacts the study coordinator (via phone number or email provided on flyer), the study coordinator will communicate via phone with the participant so that the eligibility criteria can be used to determine if the potential participant is able to be enrolled.

If the potential participant seems eligible and willing to consent to the study, the ICF and a standard medical release form will be emailed for signatures. Study coordinator will call participant to confirm consent, and complete the ICF with the potential participant. Then the baseline visit will be scheduled.

At the baseline visit, signatures will again be collected on a clean ICF in order to have study staff visualize the participant's consent to be a part of this study. After this, baseline visit procedures will occur as described in this study application.

If the potential participant prefers to meet in person for completion of the consent process, a baseline visit may still be scheduled, but no other study procedures will occur until a consent form is fully executed. This waiting period will be as long as scheduled with potential participant.

Medical Record screening for potential participants will utilize the eligibility criteria.

4. **Describe what measures will be taken to minimize the possibility of coercion or undue influence.**
ICF states that participation is voluntary. Potential participants can ask as many questions as needed.

There is no direct benefit for participation in this research project outside of the general benefits of regular exercise.

Participants will not be paid or compensated for their time and effort which also minimizes the possibility of coercion.

5. **Describe the provisions that are made to allow adequate time to exchange information and questions between the investigator and participant.**
   Potential participants will be given as much time as they need to consider the commitment to participate in this study.

   The PI will be present at the baseline visit to answer any questions or concerns above which the study coordinator can provide insight to.

6. **Will a legally authorized representative (LAR) be used?**
   ○ Yes ● **No**

7. **Will a language other than English be used to obtain consent?**
   ○ Yes ● **No**

8. **Are you requesting that documentation of informed consent be waived by the IRB (a consent process in place, but no documentation of consent, e.g. questionnaire cover letter, web-based consent, consent without signature, etc.)?**
   ○ Yes ● **No**

   If yes, complete the following:

   a. **Explain why the waiver of consent documentation is being requested.**

   b. **Justification for the waiver is one of the following:**
   There are no items to display

IRB_00122238 **Created:** 4/19/2019 11:16 AM

PI: Adriana Coletta **Submitted:** 7/1/2019

Title: Home-based high-intensity interval training to improve colorectal cancer survivorship: feasibility and relationship with novel surrogate biomarkers of colorectal cancer recurrence

IRB_00122238
5. Data Monitoring

5. Data Monitoring Plan

1. **Privacy Protections:** Privacy refers to persons and to their interest in controlling access of others to themselves. Privacy can be defined in terms of having control over the extent, timing and circumstances of sharing oneself (physically, behaviorally, or intellectually) with others. **What precautions will be used to ensure subject privacy is protected?**

   Select all that apply:

   The research intervention is conducted in a private place

   Discussing the study with participants individually instead of in front of a group

   The collection of information about participants is limited to the amount necessary to achieve the aims of the research, so that no unneeded information is being collected

   Other or additional details (specify):

2. **Confidentiality Precautions:** Confidentiality is an extension of the concept of privacy; it refers to the subject's understanding of, and agreement to, the ways identifiable information will be stored and shared. Identifiable information can be printed information, electronic information or visual information such as photographs. **What precautions will be used to maintain the confidentiality of identifiable information?**

   Select all that apply:

   Storing research data on password protected computers or in locked cabinets or offices

   Other or additional details (specify):

3. **Will photos, audio recordings, or video recordings, or medical images of participants be made during the study?**

   ◯ Yes ● **No**

   If yes, describe the recording/images and what will become of them after creation (e.g., shown at scientific meetings, stored in the medical/research record, transcribed, erased, etc.):

4. **How will study data and documentation be monitored throughout the study?**

   Select all that apply:

   Periodic review and confirmation of participant eligibility

   Periodic review of informed consent documentation

   Confirmation that all appropriate information has been reported to the sponsor, oversight agencies (such as the FDA), and/or IRB

   Other additional details (specify):

5. **Who will be the primary monitor of the study data and documentation?**

   Select all that apply:

   Principal Investigator

   Study Coordinator or Research Nurse

   Other or additional details (specify):

6. **How often is study data and documentation monitoring planned (e.g., monthly, twice a year, annually, after N participants are enrolled, etc.)?**
Annually at minimum, and after meeting enrollment goals.

IRB_00122238 **Created:** 4/19/2019 11:16 AM

PI: Adriana Coletta **Submitted:** 7/1/2019

Title: Home-based high-intensity interval training to improve colorectal cancer survivorship: feasibility and relationship with novel surrogate biomarkers of colorectal cancer recurrence

IRB_00122238
- Safety Monitoring

Safety Monitoring Plan

1. **Describe the safety monitoring entity for this study:**

   a. **Select all that apply:**

   Principal Investigator

   Please specify:

   b. **Describe the expertise and affiliation of the individual(s) selected above who will monitor the study:**
   Dr. Coletta, as PI, is an exercise physiologist with years of experience conducting cardiopulmonary exercise testing and exercise training interventions, who additionally is currently certified by the American Heart Association.

2. **Describe the data and events that will be monitored and reviewed (e.g., vital signs, safety blood labs, depression scales, neurological exams, types of adverse events, etc.):**

   Blood pressure will be monitored and reviewed at baseline visit.

   Study personnel will monitor participant adherence to HIIT and MICE workouts, notably heart rate tracking during workouts, on a weekly basis.

   Investigator response to abnormal CEA and/or ctDNA results, and EKG from cardiopulmonary exercise test:

   1. Ideally, each participant's 12-week study period will align with his/her standard of care (SOC) quarterly clinic visits; therefore the provider will know the CEA level. In the event the study period does not align with these SOC visits, if abnormal CEA is discovered, the study team will inform the patient's medical oncologist immediately upon learning of the results.

   2. ctDNA will be assessed via batch-analysis upon completion of all evaluable participants in the trial. In the event of abnormal ctDNA results, the study team will inform the patient's medical oncologist immediately upon learning of the results.

   3. In the event that an abnormal EKG is observed during the cardiopulmonary exercise test (e.g at rest preceding the start of the test, during the warm-up, during the test, or after completion of the test during the cool-down), the study team will terminate the test and inform the medical oncologist immediately upon learning of the results. Participation in the trial will be suspended for the participant until further evaluation and clearance from the medical oncologist and/or cardiologist is provided.

3. **Describe the types of reports that will be produced by the monitoring entity (e.g., safety, study progress, interim analysis, etc.):**

   No reports will be produced, but details and justification of withdrawal from study will be tracked in the RSR database on individual participants.

4. **Describe the specific triggers or stopping rules for the study:**

   a. **Under what conditions will a participant be withdrawn from the study?**
   A premature discontinuation of study will occur when a patient who signed the informed consent form ceases participation in the study, regardless of the circumstances, before the completion of the protocol.

   Patients can be prematurely discontinued from the study for one of the following reasons:

   - Screen failure

- At baseline visit, participant's resting blood pressure is greater than 140/90

- Participant requests to be withdrawn from the study

- Participant continually completes his/her HIIT or MICE workout at 100% peak heart rate

- The treating provider feels it is not in the best interest of the subject to continue participation

b. Under what conditions will the study be modified or stopped?

None.

5. How often will the data and events be reviewed by the monitoring entity (e.g., after every 5 submits, monthly, quarterly, twice a year, etc.)?

At minimum annually.

IRB_00122238 **Created:** 4/19/2019 11:16 AM

PI: Adriana Coletta **Submitted:** 7/1/2019

Title: Home-based high-intensity interval training to improve colorectal cancer survivorship: feasibility and relationship with novel surrogate biomarkers of colorectal cancer recurrence

IRB_00122238
6. Risks and Benefits

6. Risks and Benefits

1. **Describe the reasonable foreseeable risks or discomforts to the participants:**

There are some risks to participating in this study. For most of the assessments and surveys, the risk is minimal. The minimal risk includes breech of privacy and/or confidentiality.

Blood draws may cause pain, itching, burning, bruising, fainting, and may lead to an infection. Participants may faint and/or develop an infection with redness and irritation of the vein at the site where blood is drawn.

The cardiopulmonary exercise test (CPET) may cause muscle soreness, fatigue, and shortness of breath. The chance of a serious complication (such as a heart attack or death) is very low.

Exercise training (HIIT or MICE) may cause muscle soreness and/or strains, pulled muscles, joints sprains, heart problems, physical discomfort such as increased heart rate, chest pain, shortness of breath, headache, nausea, and/or fatigue, and/or accidental injuries such as falling.

In addition to above, there may be risks which are currently unknown.

2. **Describe the potential benefits to society AND to participants (do not include compensation):**

There are no anticipated direct benefits to participants. Benefits may include the known general benefits of engaging in regular exercise, such as improvements in fitness, muscular strength and endurance, and practicing self-efficacy.

The information we get from this study may help us treat future cancer patients, and may benefit society by increased understanding of how exercise may help prevent colorectal cancer recurrence in colorectal cancer survivors.

3. **Are there any costs to the participants from participation in research?**

○ Yes ● **No**

If yes, specify:

4. **Is there any compensation to the participants?**

○ Yes ● **No**

a. **If yes, answer the following:**
Specify overall amount:

b. **Specify when participants will be paid (e.g. at each visit, at end of study, etc.):**

c. **If applicable, please specify payment by visit or other time interval (e.g. $10 per visit, etc.):**

d. **If applicable, explain plan for prorating payments if participant does not complete the study:**

IRB_00122238 **Created:** 4/19/2019 11:16 AM

PI: Adriana Coletta **Submitted:** 7/1/2019

Title: Home-based high-intensity interval training to improve colorectal cancer survivorship: feasibility and relationship with novel surrogate biomarkers of colorectal cancer recurrence

IRB_00122238
7. HIPAA & the Covered Entity

7. HIPAA and the Covered Entity

1. **Does this study involve Protected Health Information (PHI) or de-identified health information?**
   ● **Yes** ○ No

   a. **Select the method(s) of authorization that will be used:**

      (Consent and) Authorization Document

      Waiver or Alteration of Authorization

   b. **Will PHI be disclosed outside the Covered Entity?**
      ○ Yes ● **No**

Does this study involve any of the following:

2. **The investigational use of a drug?**
   ○ Yes ● **No**

3. **The investigational use of a medical device?**
   ○ Yes ● **No**

4. **Is this an investigator-initiated drug or device trial lead by the Principal Investigator?**
   ○ Yes ● **No**

5. **Exposure to radioisotopes or ionizing radiation?**
   ○ Yes ● **No**

6. **A Humanitarian Device Exemption (HDE)?**
   ○ Yes ● **No**

7. **Genetic testing and/or analysis of genetic data?**
   ○ Yes ● **No**

8. **Creating or sending data and/or samples to a repository to be saved for future research uses?**
   ● **Yes** ○ No

9. **Are you:**
   - **Collecting samples of blood, organs or tissues from participants for research purposes;**
   - **Introducing Recombinant or Synthetic Nucleic Acids (e.g. viral vectors, oligonucleotides) or cells containing recombinant nucleic acids (e.g. CAR-T) into participants; OR**

- **Introducing other biological materials (e.g. bacteria, viruses) into participants.**

○ Yes ● **No**

10. **Does this study involve any of the following?**
    - **Cancer Patients**
    - **Cancer Hypothesis**
    - **Cancer risk reduction**
    - **Cancer prevention**

● **Yes** ○ No

11. **Any component of the Clinical and Translational Science Institute (CTSI)?**

○ Yes ● **No**

The Clinical Research Center (CRC)?

○ Yes ● **No**

IRB_00122238 **Created:** 4/19/2019 11:16 AM

PI: Adriana Coletta **Submitted:** 7/1/2019

Title: Home-based high-intensity interval training to improve colorectal cancer survivorship: feasibility and relationship with novel surrogate biomarkers of colorectal cancer recurrence

IRB_00122238
- Request for Waiver of Authorization

Request for Waiver or Alteration of Authorization

Request for Waiver of Authorization for Recruitment Only

This option must only be used if you are reviewing PHI in order to identify eligible participants BEFORE approaching them to obtain consent and authorization. All other waiver requests must be entered below.

Waiver of Authorization for Recruitment Requested

Other Requests for Waivers of Authorization:

- *Click "Add" below to add a new waiver request to this application.*
- *Click the waiver name link to edit a waiver that has already been created.*
- *To delete a waiver request, contact the IRB.*

Date Created	Type of Request	Purpose of Waiver Request
There are no items to display		

IRB_00122238 **Created:** 4/19/2019 11:16 AM

PI: Adriana Coletta **Submitted:** 7/1/2019

Title: Home-based high-intensity interval training to improve colorectal cancer survivorship: feasibility and relationship with novel surrogate biomarkers of colorectal cancer recurrence

IRB_00122238
Waiver of Authorization for Recruitment Only

Request for Waiver or Alteration of Authorization

Request for Waiver of Authorization for Recruitment Only

The PI must agree to the terms of this waiver request as described on this page. When the PI uses the "Submit" activity to submit the application for IRB review, a checkbox to accept the terms will be available in the "Submit" activity window.

This waiver request includes justification for waivers of consent for recruitment only, according to 45 CFR 46.116(d).

Terms for the Waiver of Authorization:

- The purpose of this waiver of authorization is to allow for the use of PHI in order to identify and recruit individuals who may be eligible to participate in the specific research described in this IRB application. The waiver of authorization is necessary to accommodate this minimal-risk research activity prior to seeking a full authorization from research participants.

- Methods for identifying individuals may include the following:
  - Reviewing medical charts
  - Reviewing databases that include PHI
  - Reviewing other medical- or health-based documents that include PHI

- Identifiable information used under this waiver may include the following, as this is the minimum necessary for identifying eligible individuals:
  - Name
  - Contact information, such as phone number, address, or email address
  - An ID number, such as MRN or SSN
  - Date of birth
  - Medical and health information that may determine study eligibility

- Any PHI recorded by the study team will only be used for recruitment and determining study eligibility. After this has been completed, the PHI must be removed from the research record or destroyed, unless the participants have given authorization for continued use of the PHI.

- PHI will only be viewed by approved members of the study team and will not be disclosed for research purposes to any individual or institution without the participants' authorization for such use and disclosure of the PHI.

- PHI will be stored in a secure manner according to HIPAA privacy and security provisions.

IRB_00122238 **Created:** 4/19/2019 11:16 AM

PI: Adriana Coletta **Submitted:** 7/1/2019

Title: Home-based high-intensity interval training to improve colorectal cancer survivorship: feasibility and relationship with novel surrogate biomarkers of colorectal cancer recurrence

IRB_00122238
- Data & Tissue Banking

Data & Tissue Banking

1. **Select the items that will be banked:**
   - [x] **Biological samples**
   - [ ] Data

   Type(s) of samples to be collected:
   Blood plasma

2. **What type(s) of future research will be allowed on the data/samples?** Samples will be saved for future research to learn about, prevent, and/or treat cancer.

3. **Who manages the repository and where will the data/samples be stored?**
   The Huntsman Cancer Institute Biorepository and Molecular Pathology (BMP) Director, located on the 3rd floor of HCI Research North.

4. **Indicate whether the data/samples in the repository will be identifiable directly or through a code/link.**

   a. **Select one of the following options:**
   OPTION 1: All data/samples will be identifiable to one or more individuals who have responsibilities to manage or oversee the repository.

   b. **If you selected OPTION 1 or 2 above, describe the process for managing the identifiable data:**

   ### Who will manage and have access to the identifiable data?
   The PI, Study Coordinators, and BMP staff will have access to the identifiable data.

   ### Where will the data be kept?
   Sample information, including PHI, obtained for this study will be appropriately managed through the Integrative Transdisciplinary Biospecimen Pathology Annotation and Translational Health (itBioPath) database through BMP. Clinical, patient information and other relevant information (including PHI) obtained for this study will be appropriately managed through the Research Subject Registry (RSR) database, which can link with ItBioPath.

   HIPAA guidelines are strictly followed to protect patient privacy. Both the itBioPath and RSR databases are used for de-identification of samples; itBioPath tracks sample use, amounts, and transformations from the original sample and RSR tracks information collected for the study.

   ### How will the data be kept confidential?
   Biospecimens will be labeled with specimen identification numbers, not with patient names

   As samples are brought to BMP, the BMP staff will provide participant sampels with a unique identifier. The itBioPath database is used for de-identification and safe harbor of PHI in this study; itBioPath tracks sample use, amounts, and transformations from the original sample. The itBioPath has a strong yet flexible security model to protect identifying information from unauthorized access. The security model restricts access according to the user's authorized protocols and projects.

   Only authorized individuals with HIPAA training are allowed to view PHI.

5. **Describe the procedures for participants to withdraw their data/samples from the repository. If participants will not be able to withdraw their samples, please provide an explanation:**
   Participants will notify the study coordinator or (PI) that they wish to withdraw their samples.

6. **Will future research results or findings be communicated to the participants?**
   ○ Yes ● **No**

7. **Describe the procedures for other researchers to obtain data/samples from the repository for use in future research.**

   Other researchers must request these samples from the PI. The requesting researcher must document that their research falls under an IRB approved protocol and is cancer related. If adequate samples are available, the research question is valid, and the PI approves, samples will be provided to the applying researcher. Distribution will be recorded in the Research Subject Registry database. If researchers are outside the University of Utah, an MTA will be required.

IRB_00122238 **Created:** 4/19/2019 11:16 AM

PI: Adriana Coletta **Submitted:** 7/1/2019

Title: Home-based high-intensity interval training to improve colorectal cancer survivorship: feasibility and relationship with novel surrogate biomarkers of colorectal cancer recurrence

8. Resources and Responsibilities

1. * **State and justify the qualifications of the study staff:**

Study Coordinators - are up-to-date on CITI trainings and have taken other research support classes available through the University of Utah Research Administration Training Series. Will support in the consent process, communication, data entry, data management, and administrative support.

Statistician - involved with the Cancer Biostatistics Shared Resource at HCI; will conduct the quantitative analyses.

SKAGGs Personnel - Staff at the L.S. Skaggs Patient Wellness Center will assist with the facilities for the baseline and end of study visits - mainly for the various procedures in conjuncture with the exercise equipment.

Intermountain Healthcare (IHC) - Mark Lewis, MD will refer patients from the IHC system, by providing the study flyer. He may be supported by Navigators, who are staff that are involved with and specialize in GI cancer survivorship.

2. * **Describe the training that study staff and investigators will receive in order to be informed about the protocol and understand their research-related duties and functions:**

Regular meetings with the PI to discuss enrollments, study progress and all research related progress or needs.

3. * **Describe the facilities where the research activities will be performed (e.g. hospitals, clinics, laboratories, classrooms/schools, offices, tissue banks, etc.).**

Locations within the University of Utah's covered entity - hospitals, clinics, research buildings, and exercise labs.

For communication and data maintenance: Huntsman Cancer Institute

For study procedures with participants: SKAGG Wellness Center, Health Education Professional Building (HPEB).

4. * **Describe the medical or psychological resources available at this site (and other participating sites, if applicable) that participants might require as a consequence of the research. If not applicable, please state.**

Participants may visit their primary care providers or access emergency medical care if needed for research related injury.

IRB_00122238 **Created:** 4/19/2019 11:16 AM

PI: Adriana Coletta **Submitted:** 7/1/2019

Title: Home-based high-intensity interval training to improve colorectal cancer survivorship: feasibility and relationship with novel surrogate biomarkers of colorectal cancer recurrence

IRB_00122238
Documents and Attachments

Documents and Attachments

If any of your documents (such as investigational brochures, sponsor protocols, advertisements, etc.) are not available in an electronic format, please scan and save them as PDF files or contact our office for assistance.

Naming Documents: Please use the title field to clearly indicate the content of each form. The name you enter will be listed on your approval letter. Use names that will differentiate from earlier versions.

Examples:
Consent Document Control Group 04/14/05
Consent Document Treatment Group 4/14/05
Sponsor Protocol 04/14/05 Version 2
Assent Document(Highlighted Changes)

Apple/Macintosh Users:MS Word documents must have a .doc file extension. See ERICA home page for instructions.

Print View: IRB Draft Protocol Summary

eProtocol Summary:

Name	Version	Date Created	Date Modified	Date Approved
There are no items to display				

Consent Documents, Consent Cover Letters, Consent Information Sheets, Consent Scripts, etc.:

Name	Version	Date Created	Date Modified	Date Approved
There are no items to display				

Parental Permission Documents:

Name	Version	Date Created	Date Modified	Date Approved
There are no items to display				

Assent Documents:

Name	Version	Date Created	Date Modified	Date Approved
There are no items to display				

VA Consent Documents:

Name	Version	Date Created	Date Modified	Date Approved
There are no items to display				

Surveys, Questionnaires, Interview Scripts, etc.:

Name	Version	Date Created	Date Modified	Date Approved
There are no items to display				

Full Protocol (company protocol, sponsor protocol, investigator-initiated protocol, etc.):

Name	Version	Date Created	Date Modified	Date Approved
There are no items to display				

Investigational Brochure (IB) for Investigational Drug or Drug/Device Package Insert:

Name	Version	Date Created	Date Modified	Date Approved

There are no items to display

Literature Cited/References:

Name	Version	Date Created	Date Modified	Date Approved

There are no items to display

Principal Investigator's Scholarly Record (CV/Resume):

Name	Version	Date Created	Date Modified	Date Approved
Adriana Coletta CV(0.01)	0.01	10/6/2020 3:06 PM	10/6/2020 3:06 PM	
Adriana Coletta Updated CV(0.01)	0.01	7/7/2022 2:10 PM	7/7/2022 2:10 PM	

Faculty Sponsor's Scholarly Record (CV/Resume):

Name	Version	Date Created	Date Modified	Date Approved

There are no items to display

Other Stamped Documents:

Name	Version	Date Created	Date Modified	Date Approved

There are no items to display

Recruitment Materials, Advertisements, etc.:

Name	Version	Date Created	Date Modified	Date Approved

There are no items to display

Other Documents:

Name	Version	Date Created	Date Modified	Date Approved

There are no items to display

Date: 4/19/2019 11:16 AM

Principal Investigator: Adriana Coletta

Department: Kinesiology Program

Site Staff and Enrollment:

Site Name	Investigator	Staff	Enrollment Number
University of Utah	Adriana Coletta	Glynn Gilcrease<br>Allie Memmott<br>Emily Dunston<br>J. Robinson Singleton<br>Ann Marie Moraitis<br>Benjamin Haaland<br>Kimberly Norman<br>Kristin Barber<br>Benjamin Solomon<br>Paula Hobson<br>Ignacio Garrido-Laguna	up to 50

Total number of subjects enrolled at the University of Utah in all years prior to October 2019: up to 50

Title: Home-based high-intensity interval training to improve colorectal cancer survivorship: feasibility and relationship with novel surrogate biomarkers of colorectal cancer recurrence

---

The PRMC is charged with the review of the scientific merit, priorities, and progress of cancer research at the University of Utah.

What type of Clinical Research is this?

Patient-oriented research: This type of research is conducted with human subjects (or on material of human origin such as tissues, specimens and cognitive phenomena) for which an investigator (or colleague) directly interacts with human subjects. Patient-oriented research includes:
- Studies of mechanisms of human disease
- Studies of therapies or interventions for disease
- Clinical trials, and
- Studies to develop new technology related to disease

Multicenter Study:

No

Coordinating Center or Organization:

PSTO

Project Funding:

	Sponsor	Sponsor Type	Sponsor Contact Information	Prime Sponsor	Prime Sponsor Type	OrgID
View	HUNTSMAN CANCER INSTITUTE	UofU	Adriana Coletta's Research Start-up Funds and Supportive Oncology and Survivorship Center Pilot Project Award			215

1. **Clinical Research Category (Select One):**
   Interventional (INT)

2. **Primary Purpose of the Research (Select One):**
   Supportive Care (SUP)

3. **Study Source (Select One):**
   Institutional: This category may include
   a. Single or Multi-Institutional studies authored and implemented by investigators at the University of Utah (Note: National and externally peer-reviewed studies should be listed with those categories, not as Institutional studies)
   b. Institutional studies authored and implemented by investigators at another Center in which the University of Utah is participating
   c. Institutional cancer projects will be evaluated for HCI PRMC approval prior to IRB submission.

Does the protocol describe the data and safety monitoring plan (required for institutional treatment trials):
Yes

Will the study be monitored (for data and safety) at HCI or by another organization:
Yes

Name of data and safety monitoring organization:
RCO

Safety monitoring entity, expertise and affiliation:

Principal Investigator

Dr. Coletta, as PI, is an exercise physiologist with years of experience conducting cardiopulmonary exercise testing and exercise training interventions, who additionally is currently certified by the American Heart Association.

Specific triggers or stopping rules for individual participants:
A premature discontinuation of study will occur when a patient who signed the informed consent form ceases participation in the study, regardless of the circumstances, before the completion of the protocol.

Patients can be prematurely discontinued from the study for one of the following reasons:

- Screen failure
- At baseline visit, participant's resting blood pressure is greater than 140/90
- Participant requests to be withdrawn from the study
- Participant continually completes his/her HIIT or MICE workout at 100% peak heart rate

- The treating provider feels it is not in the best interest of the subject to continue participation

Specific triggers or stopping rules for entire study:

None.

4. **Have project statistics been reviewed by a statistician:**
   Yes

5. **How will accrual and demographic information be tracked:**
   Research Subject Registry (RSR)

6. **Does this study target a Rare Disease (meaning it targets a cancer with an incident rate of = < 3 per 100,000 per year):**
   ○ Yes ● **No**

7. **Number of subjects to be enrolled at the University of Utah in year 1:**
   30

8. **Estimated number of potentially eligible subjects seen at the University in the past year:**
   70

9. **Source of data (e.g. CTRG estimate, cancer registry, clinic stats, TriNetX etc.):**
   CCR Colon Database

10. **Estimated time (in months) for full accrual at the University of Utah:**
    24

11. **Is this study run through the HCI Clinical Trials Office:**
    No

12. **Department overseeing this research:**
    HCI Population Sciences Trials Office (PSTO)

Comments:

IRB_00122238 **Created:** 4/19/2019 11:16 AM

PI: Adriana Coletta **Submitted:** 7/1/2019

Title: Home-based high-intensity interval training to improve colorectal cancer survivorship: feasibility and relationship with novel surrogate biomarkers of colorectal cancer recurrence

IRB_00122238
IRB Smart Form - Read Only

Sponsor Information

a. **Sponsor:**
HUNTSMAN CANCER INSTITUTE

b. **Sponsor Contact Information:**
Address, phone number, fax number used for event reporting and study correspondence.

Adriana Coletta's Research Start-up Funds and Supportive Oncology and Survivorship Center Pilot Project Award

c. **If the funding type is "Federal Agency, or federal flow through", provide the following information:**

Grant Number:

Grant Awardee (Institution and Investigator):

Effective Start Date:

Effective End Date:

If the grant has been awarded to the University of Utah, please attach a copy of the grant application to the Documents and Attachments page.

d. **Are you working on this study with the University of Utah Office of Sponsored Projects to obtain this funding?**
No

If no, please explain:

A contract with the Office of Sponsored Projects may be required even if you are not receiving direct funds. Any relationship with an outside entity should be discussed with OSP:

Main Campus OSP: ***801.581.6903***
UUHSC OSP: ***801.581.8949***
VAMC OSP: ***801.582.1565 x4866***

IRB_00122238 **Created:** 4/19/2019 11:16 AM

PI: Adriana Coletta **Submitted:** 7/1/2019

Title: Home-based high-intensity interval training to improve colorectal cancer survivorship: feasibility and relationship with novel surrogate biomarkers of colorectal cancer recurrence

Finish Instructions

Finish Instructions

1. **To view errors, select the "Validate" option at the top-left of the page. If you have errors on your application, you won't be able to submit it to the IRB.**
2. **Selecting the Finish button will NOT submit the application to the IRB.**
   You MUST select the "Submit" option on the workspace once you've selected the "Finish" button.
3. **If your study has a faculty sponsor: Once the PI submits the application, it will be sent to the faculty sponsor for final approval. The IRB cannot review the study until the faculty sponsor submits the application to the IRB.**
